# Exploring potential risk factors for lower limb amputation in people with diabetes—A national observational cohort study in Sweden

**DOI:** 10.1002/jfa2.70005

**Published:** 2024-09-01

**Authors:** Simon Ramstrand, Michael Carlberg, Gustav Jarl, Anton Johannesson, Ayako Hiyoshi, Stefan Jansson

**Affiliations:** ^1^ Faculty of Medicine and Health University Health Care Research Center Örebro University Örebro Sweden; ^2^ Department of Rehabilitation School of Health Sciences Jönköping University Jönköping Sweden; ^3^ Clinical Epidemiology and Biostatistics Faculty of Medicine and Health Örebro University Örebro Sweden; ^4^ Össur Clinics Scandinavia Stockholm Sweden; ^5^ Department of Public Health and Caring Sciences Uppsala University Uppsala Sweden

**Keywords:** amputation, diabetes, diabetic foot, register study, risk factors

## Abstract

**Aims:**

Risk factors for lower limb amputation (LLA) in individuals with diabetes have been under‐studied. We examined how 1/demographic and socioeconomic, 2/medical, and 3/lifestyle risk factors may be associated with LLA in people with newly diagnosed diabetes.

**Methods:**

Using the Swedish national diabetes register from 2007 to 2016, we identified all individuals ≥18 years with an incident diabetes diagnosis and no previous amputation. These individuals were followed from the date of diabetes diagnosis to amputation, emigration, death, or the end of the study in 2017 using data from the In‐Patient Register and the Total Population Register. The cohort consisted of 66,569 individuals. Information about demographic, socioeconomic, medical, and lifestyle risk factors was ascertained around the time of the first recorded diabetes diagnosis, derived from the above‐mentioned registers. Cox proportional hazard models were used to obtain hazard ratios (HR) with 95% confidence intervals (CI).

**Results:**

During the median follow‐up time of 4 years, there were 133 individuals with LLA. The model adjusting for all variables showed a higher risk for LLA with higher age, HR 1.08 (95% CI 1.05–1.10), male sex, HR 1.57 (1.06–2.34), being divorced, HR 1.67 (1.07–2.60), smokers HR 1.99 (1.28–3.09), insulin treated persons HR 2.03 (1.10–3.74), people with low physical activity (PA) HR 2.05 (1.10–3.74), and people with an increased foot risk at baseline HR > 4.12. People with obesity had lower risk, HR 0.46 (0.29–0.75).

**Conclusions:**

This study found a higher risk for LLA among people with higher age, male sex, who were divorced, had a higher foot risk group, were on insulin treatment, had lower PA levels, and were smokers. No significant association was found between risk for LLA and education level, country of origin, type of diabetes, blood glucose level, hypertension, hyperlipidemia, creatinine level, or glomerular filtration rate. Obesity was associated with lower risk for LLA. Identified variables may have important roles in LLA risk among people with diabetes.

## 1 INTRODUCTION

Diabetes is a severe, chronic condition affecting 537 million people worldwide and causing premature death, disability, and economic loss [1]. In Sweden, the prevalence of diabetes is approximately 5.5% and the economic burden of complications in type 2 diabetes is substantial [2, 3].

Diabetes‐related foot ulcers (DFUs) are the most common cause of nontraumatic amputations of the lower limbs in the Western world [4] and are one of the most expensive complications of diabetes [5, 6]. Although people with DFU undergo lower limb amputations (LLA) at a relatively young age—the median age is approximately 65 years—the 5‐year mortality rate in people with DFU is high and comparable to that of individuals diagnosed with colon cancer [7]. Furthermore, the risk for LLA is 8–20 times higher for individuals with diabetes than for those without diabetes [8]. LLA in people with diabetes has also been shown to be a major cause of disability, both physical and mental, and is associated with a decreased quality of life [9, 10]. Studies examining temporal changes in incidence rates for different levels of LLA among individuals with diabetes have remained inconclusive. The incidence rate of major amputations generally decreases over time, but the direction of change in the incidence rates of minor amputations varies [11, 12, 13, 14].

Exploring risk factors that may be associated with a higher risk of LLA in individuals with diabetes is important to identify interventions that focus on known risk factors with a strong association with LLA. Previous studies have examined some demographic and socioeconomic, medical, and lifestyle risk factors and their association with LLA [15, 16, 17]. Risk factors that have been identified as having an association with LLA include hyperglycemia, hyperlipidemia, hypertension, previous revascularization, peripheral arterial disease, presence of a walking disability, and poor attendance to care. It is important to recognize however that these studies focused on groups in which DFUs are already present. In guidelines from the international workgroup on the diabetes foot (IWGDF), physical/medical factors are lifted as indicators of ulcer risk. The group specifically mentions loss of protective sensation and peripheral artery disease as risk factors in persons with later stage diabetes [18]. Knowledge regarding if and how risk factors differ for persons with earlier‐stage diabetes and without DFU is limited. There is a need to determine if general risk factors apply to persons without DFU and, if there are specific risk factors that should be addressed differently in persons without DFU. This knowledge will pave the way for development of appropriately targeted interventions which can be implemented prior to DFUs being established.

In the present study we aimed to explore how demographic and socioeconomic, medical, and lifestyle risk factors are associated with subsequent LLA in persons with newly diagnosed diabetes.

## 2 METHODS

### 2.1 Study design and data

We conducted a total population explorative cohort study using four Swedish national registers. Every Swedish citizen has a unique 12‐digit identification number which is included in all register data used in this study and enables it to be linked across registers at the individual level [19]. Based on data from the National Diabetes Register (NDR), we identified all individuals 18 years of age or older with a registered incident diagnosis of diabetes between 2007 and 2016. Health care providers in primary healthcare centers and hospital outpatient clinics in Sweden report information on diabetes patients' medical and lifestyle variables to the NDR [20]. In most clinics, data is transferred to the NDR by direct data transmission from electronic medical records. The NDR currently has a national coverage of 97% for patients with type 1 diabetes and 87% for patients with type 2 diabetes. Demographic and socioeconomic variables were obtained from the longitudinal integrated database for health insurance and labor market studies (LISA), which compiles data by calendar year for the total adult Swedish population aged ≥16 years [21, 22]. Inclusion of personal data in Swedish government‐administered registers such as LISA is mandatory. Data regarding occupation has a completeness of 95% in the LISA registry while data related to education has a completeness of >98% for all individuals aged between 25 and 64 years. Income data primarily consists of income from employment, capital, and allowances, including parental allowance. In Sweden, work force participation is around 80% [23]. Information on LLA was obtained from the inpatient register (IPR) [24], part of the National Patient Register. The IPR has been recognized as having high validity and complete national coverage since 1987 (See Supporting Information S1: Electronic Supplement). In the IPR, diagnoses are recorded using the Swedish version of the International Classification of Disease (ICD‐10) system, adapted from the World Health Organization ICD classification system. Finally, we obtained dates of emigration and death from the Total Population Register where virtually 100% of births and deaths, 95% of immigrations and 91% of emigrations are reported within 30 days, with a higher proportion over time [25]. Using these data, individuals were followed from the time of the first recorded diabetes diagnosis and ending on the date of LLA, emigration, death, or on the December 31, 2017, whichever came first.

### 2.2 Variables

#### 2.2.1 Demographic and socioeconomic, medical, and lifestyle risk factors

The following variables were analyzed as potential risk factors: age, sex, country of origin (born in Sweden/outside Sweden), marital status, education, diabetes type, diabetes drug treatment, hemoglobin A1c, hypertension, hyperlipidemia, creatinine level, glomerular filtration rate (GFR), foot risk group (1. Healthy foot, 2. Neuropathy/angiopathy, 3. Previous DFU, and 4. Ongoing severe foot disease), body mass index (BMI), smoking, and physical activity (PA). We selected these variables because they were included in the NDR, indicating their importance in diabetes management, and had less than 40% missing data. An exception from the 40% cut off was the foot risk group, which is clinically important and had marginally more than 40% of data missing (40.8%). Hypertension and hyperlipidemia were defined based on the presence of ongoing drug treatment for these conditions. All variables, except temporal data related to amputation, are presented with values for the same year as the diabetes diagnosis. The last entry was used in subjects with more than one data entry in the year of diagnosis. The variables were divided into three groups, which were used to present results: demographic and socioeconomic variables, medical variables, and lifestyle variables.

#### 2.2.2 Outcome

We identified individuals who had an LLA through the IPR and then cross‐referenced the data with the NDR to identify persons with diabetes. The date of incident (lifetime first) LLA amputation was identified using operation codes recorded in the IPR using the Nordic Classification of Surgical Procedures codes (for full information see Supporting Information S1: Electronic Supplement):

1) High proximal amputations (codes NEQ19, NFQ09, NFQ19, and NGQ09),
2) Low proximal amputations (NGQ19, NHQ09, and NHQ11), and
3) Partial foot amputations (NHQ12, NHQ13, and NHQ14)

Use of the procedural codes meant that all LLAs except toe amputations were identified. A re‐amputation from the toe level to a more proximal level was subsequently considered an initial amputation. If bilateral amputations were performed on the same date, only one amputation was used (either the most proximal amputation or one of the legs if both were amputated at the same level).

### 2.3 Study population

**FIGURE 1:**
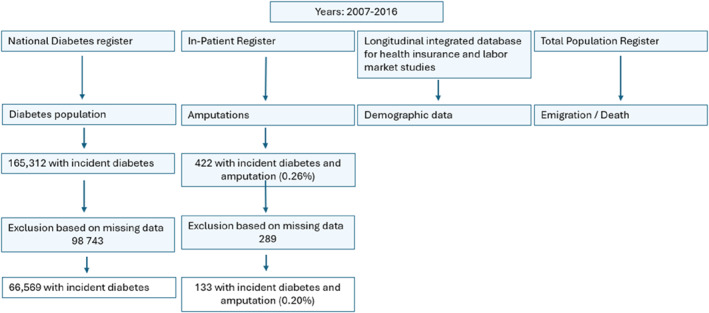
Registers included, study population, and exclusions. All participants were originally identified through the national diabetes register.

Among the 165,312 individuals with diabetes identified between 2007 and 2016, 422 had an LLA. After excluding variables with more than 40% missing data, we excluded persons with incomplete data on the included variables of interest. The final cohort consisted of 66,569 individuals, of whom 133 had an amputation (Figure 1). Of these, a small proportion (2%) had type 1 diabetes; therefore, these individuals did not significantly influence the results.

The study was approved by the regional ethics committee review board in Uppsala, Sweden (reg. no. 2017/533 and 2017/533/1).

### 2.4 Statistical methods

Descriptive statistics were used to summarize demographic and socioeconomic, medical, and lifestyle characteristics at baseline and at first recorded diabetes diagnosis. For analysis of included variables, a *t*‐test and chi‐square test were used to compare the distribution between persons who had an LLA and those who did not during follow‐up. Cox proportional hazards regression analysis was used for survival analysis and to calculate hazard ratios (HR) (HRs) with 95% confidence intervals (CI) (CIs). We examined the proportional hazards assumption using Schoenfeld residuals and no evidence of violation was found. Unadjusted models and a model mutually adjusting for all variables were fitted, where the adjusted model was used to control for codependences between variables. *p*‐values <0.05 were considered statistically significant. Analyses were conducted using Stata/MP 15.1.

## 3 RESULTS

### 3.1 Comparison of people with and without LLA

The study population consisted of 66,569 individuals who were diagnosed with diabetes, approximately 98% of whom had type 2 diabetes between 2007 and 2016 (Table 1). During a median follow‐up time of 4 years, 133 (0.2%) participants underwent an LLA. The incidence rate of LLA was 44.2 annual cases (95% CI 37.3–52.4) per 100,000 individuals with diabetes.

**TABLE 1:** Baseline.

	*N* amputation (*n* = 66,436)	Amputation (*n* = 133)	
	*N* or mean^a^	*N*	*p*‐value^b^
Demographic and socioeconomic variables			
Age			<0.001
Mean (SD)	63.6 (11.4)	72.6 (10.5)	
Gender			0.12
Men, *n* (%)	38,574 (58.1)	86 (64.7)	
Women, *n* (%)	27,862 (41.9)	47 (35.3)	
Country of origin			0.02
Other, *n* (%)	12,765 (19.2)	15 (11.3)	
Sweden, *n* (%)	53,671 (80.8)	118 (88.7)	
Marital status			<0.001
Married, *n* (%)	35,510 (53.4)	58 (43.6)	
Single, *n* (%)	12,074 (18.2)	16 (12)	
Divorced, *n* (%)	12,463 (18.8)	31 (23.3)	
Widowed, *n* (%)	6389 (9.6)	28 (21.1)	
Education^c^			0.002
High, *n* (%)	13,427 (20.2)	18 (13.5)	
Mid, *n* (%)	30,595 (46.1)	51 (38.3)	
Low, *n* (%)	22,414 (33.7)	64 (48.1)	
Medical variables			
Diabetes type			0.65
Type 1, *n* (%)	1369 (2.1)	2 (1.5)	
Type 2, *n* (%)	65,067 (97.9)	131 (98.5)	
Diabetes treatment			0.002
Diet only, *n* (%)	26,468 (39.8)	60 (45.1)	
Tablets, *n* (%)	33,955 (51.1)	49 (36.8)	
Insulin, *n* (%)	2885 (4.3)	15 (11.3)	
Tablets + insulin, *n* (%)	2955 (4.4)	9 (6.8)	
Injection (GLP‐1), *n* (%)	174 (0.3)	0 (0)	
Blood glucose level HbA1c			0.007
Mean, % NGSP (SD)	6.7 (3.4)	7.0 (3.6)	
Mean, mmol/mol IFCC (SD)	50.1 (13.1)	53.2 (15.7)	
Hypertension			0.01
No, *n* (%)	21,795 (32.8)	30 (22.6)	
Yes, *n* (%)	44,641 (67.2)	103 (77.4)	
Hyperlipidemia			0.37
No, *n* (%)	35,881 (54)	77 (57.9)	
Yes, *n* (%)	30,555 (46)	56 (42.1)	
Creatinine (mg/mmol)			<0.001
Mean (SD)	76 (22.4)	86.9 (35.0)	
Glomerular filtration rate (mL/min, 1.73 m^2^)			<0.001
Mean (SD)	85.5 (22.7)	76.8 (26.7)	
Foot risk group			<0.001
1. Healthy foot (diabetes without foot complication), *n* (%)	57,586 (86.7)	65 (48.9)	
2. Neuropathy and/or angiopathy, *n* (%)	8250 (12.4)	57 (42.9)	
3. Previous diabetes foot ulcers (incl. Amputation), *n* (%)	331 (0.5)	5 (3.8)	
4. Ongoing severe foot disease^d^, *n* (%)	270 (0.4)	6 (4.5)	
Lifestyle variables			
Body mass index (kg/m^2^)			<0.001
Mean (SD)	30.4 (5.6)	28 (5.3)	
Body mass index (kg/m^2^), groups			<0.001
Underweight (15.0–<18.5), *n* (%)	177 (0.3)	2 (1.5)	
Normal weight (18.5–<25.0), *n* (%)	9584 (14.4)	35 (26.3)	
Overweight (25.0–<30.0), *n* (%)	25,320 (38.1)	55 (41.4)	
Obese (≥30.0), *n* (%)	31,355 (47.2)	41 (30.8)	
Smoking			0.04
No, *n* (%)	56,296 (84.7)	104 (78.2)	
Yes, *n* (%)	10,140 (15.3)	29 (21.8)	
Physical activity			0.001
Low activity, <1 time/week, *n* (%)	15,330 (23.1)	52 (39.1)	
Regular, 1–5 times/week, *n* (%)	29,213 (44)	48 (36.1)	
Daily, *n* (%)	21,893 (33)	33 (24.8)	

*Note*: Distribution, no amputation/amputation for individuals with complete data on the included variables.

Abbreviations: IFSC, International Federation of Clinical Chemistry; NGSP, National Glycohemoglobin Standardization Program.

^a^ Categorical values are *n* (%) and continues values are mean (SD).

^b^ Chi‐squared or *t*‐test.

^c^ Education levels are defined as: Low: elementary school (≤9 years), Mid: Secondary education (2–3 years), High: Post‐secondary education.

^d^ Ongoing severe foot disease according to NDR includes: Diabetic foot ulcer, critical ischemia, infection, Charcot foot.

Regarding demographic and socioeconomic variables at baseline, that is, at the time of diabetes diagnosis, individuals who later had an LLA were older (mean 72.6 years) and had a higher rate of being divorced (23.3% vs. 18.8%) or widowed (21.1% vs. 9.6%).

In the medical category, significant associations were found between LLA and diabetes treatment, where the group who had LLA used “diet only” to a higher extent (45.1% vs. 39.8%), received insulin more (11.3% vs. 4.3%) and tablets less (36.8% vs. 51.1%). A significant association was also found for blood glucose level (HbA1c). Participants who later had an LLA were more likely to be drug treated for hypertension (77.4% vs. 67.2%), had higher creatinine levels (mean of 35.0 vs. 22.4), had lower GFR scores (mean of 27.7 vs. 22.7) and were less likely to have healthy feet (48.9% vs. 86.7%) at time of diagnosis. Regarding foot status, of those who had foot risk category 1 at time of diagnosis 0.1% (65 out of 57,586) later had an LLA, for category 2 0.7% (57 out of 8250) later had an LLA, for foot risk category 3 1.5% (5 out of 331) later had an LLA and for foot risk category 4 2.2% (6 out of 270) later had an LLA.

For the lifestyle category, those who later had an LLA were less likely to be obese (30.8% vs. 47.2%) and were more likely to have normal weight (26.3% vs. 14.4%) at the time of diagnosis. Those who later had an LLA were more likely to be smokers (21.8% vs. 15.3%) and had lower PA (39.1% vs. 23.1 for low activity) at the time of diabetes diagnosis than those who would not be amputated.

### 3.2 Associations between potential risk factors and LLA

In the unadjusted model presented in Table 2, a statistically significant higher risk of LLA was observed regarding demographic and socioeconomic variables for individuals who were older, HR 1.09 (95% CI 1.07–1.11), were divorced, 1.65 (1.07–2.55) or widowed, 2.83 (1.80–4.45) compared to being married and, had a low education level 1.94 (1.15–3.27) compared with high education level.

**TABLE 2:** Demographic and socioeconomic variables—Results of Cox proportional hazards regression analysis for association with LLA.

	Amputated/N	Unadjusted HR	Adjusted HR (95% CI)
Age (continuous variable)		1.09 (1.07–1.11)	1.08 (1.05–1.10)
Gender			
Men	86/38,660	Ref	Ref
Women	47/27,909	0.74 (0.52–1.05)	**0.64 (0.43–0.95)**
Born in Sweden			
Yes	118/53,789	Ref	Ref
No	15/12,780	0.63 (0.37–1.07)	0.90 (0.52–1.56)
Marital status			
Married	58/35,568	Ref	Ref
Single	16/12,090	0.85 (0.49–1.47)	1.19 (0.67–2.12)
Divorced	31/12,494	**1.65 (1.07–2.55)**	**1.67 (1.07–2.60)**
Widowed	28/6417	**2.83 (1.80–4.45)**	1.40 (0.85–2.29)
Education			
High	18/13,445	Ref	Ref
Mid	51/30,646	1.18 (0.69–2.02)	1.10 (0.64–1.90)
Low	64/22,478	1.94 (1.15–3.27)	1.13 (0.66–1.93)

*Note*: Values in bold text marks statistical significance.

Abbreviations: CI, confidence interval; HR, hazard ratio; LLA, lower limb amputation; SD, standard deviation.

Among medical variables (Table 3), there was an increased risk of LLA for those who received insulin for diabetes treatment, 2.33 (1.32–4.10), had hypertension, 1.72 (1.14–2.58), had lower GFR, (continuous variable) 0.98 (0.97–0.99) and had a poorer foot risk category compared to healthy feet, 7.44 (5.21–10.63).

**TABLE 3:** Medical variables—Results of Cox proportional hazards regression analysis for association with LLA.

	Amputated/N	Unadjusted HR	Adjusted HR (95% CI)
Diabetes type			
Type 1	2/1371	0.59 (0.15–2.38)	0.66 (0.15–2.81)
Type 2	131/65,198	Ref	Ref
Diabetes treatment			
Diet only	60/26,528	Ref	Ref
Tablets	49/34,004	0.80 (0.55–1.17)	1.01 (0.68–1.50)
Insulin	15/2899	**2.33 (1.32–4.10)**	**2.03 (1.10–3.74)**
Tablets + insulin	9/2964	1.50 (0.74–3.02)	1.69 (0.81–3.53)
Injection (GLP‐1)	0/174	–	–
Blood glucose level HbA1c (mmol/mol; continuous variable)		1.01 (1.00–1.02)	1.01 (1.00–1.03)
Hypertension			
No	30/21,825	Ref	Ref
Yes	103/44,744	**1.72 (1.14–2.58)**	1.23 (0.79–1.90)
Hyperlipidemia			
No	77/35,958	Ref	Ref
Yes	56/30,611	0.85 (0.61–1.20)	0.76 (0.53–1.09)
Creatinine (mg/mmol; continuous variable)		1.00 (1.00–1.01)	1.00 (1.00–1.01)
Glomerular filtration rate (mL/min; continuous variable)		**0.98 (0.97–0.99)**	1.00 (0.99–1.01)
Foot risk category			
Healthy foot (diab w/o complication)	65/57,650	Ref	Ref
Neuropathy, angiopathy	57/8307	**7.44 (5.21–10.63)**	**4.12 (2.84–5.98)**
Previous diabetes wounds (incl.amputation)	5/336	**15.18 (6.11–37.69)**	**8.26 (3.29–20.74)**
Ongoing severe foot disease^a^	6/276	**14.89 (6.45–34.39)**	**11.24 (4.82–26.23)**

*Note*: Values in bold text marks statistical significance.

Abbreviations: CI, confidence interval; HR, hazard ratio; LLA, lower limb amputation; SD, standard deviation.

^a^ Ongoing severe foot disease according to NDR includes: Diabetic foot ulcer, critical ischemia, infection, Charcot foot.

Regarding lifestyle variables (Table 4), those who were underweight 4.22 (1.02–17.57), smoked 1.58 (1.05–2.39), or had a low PA level compared to daily PA 2.67 (1.72–4.13) had an increased risk. There were also two variables indicated as being protective factors, that is, associated with decreased risk for LLA, namely people being overweight 0.56 (0.37–0.86) or obese 0.34 (0.22–0.54) had a lower risk of LLA than people with normal weight.

**TABLE 4:** Lifestyle variables—Results of Cox proportional hazards regression analysis for association with LLA.

	Amputated/N	Unadjusted HR	Adjusted HR (95% CI)
Lifestyle variables			
Body mass index (kg/m^2^), groups			
Underweight (15.0–<18.5)	2/179	**4.22 (1.02–17.57)**	3.48 (0.82–14.75)
Normal weight (18.5–<25.0)	35/9619	Ref	Ref
Overweight (25.0–<30.0)	55/25,375	**0.56 (0.37–0.86)**	0.65 (0.42–1.00)
Obese (≥30.0)	41/31,396	**0.34 (0.22–0.54)**	**0.46 (0.29–0.75)**
Smoking			
No	104/56,400	Ref	Ref
Yes	29/10,169	**1.58 (1.05–2.39)**	**1.99 (1.28–3.09)**
Physical activity			
Low activity, <1 time/week	52/15,382	**2.67 (1.72–4.13)**	**2.05 (1.30–3.23)**
Regular, 1–5 times/week	48/29,261	1.08 (0.70–1.69)	1.14 (0.73–1.79)
Daily	33/21,926	Ref	Ref

*Note*: Values in bold text marks statistical significance.

Abbreviations: CI, confidence interval; HR, hazard ratio; LLA, lower limb amputation; SD, standard deviation.

In the adjusted model, when considering demographic and socioeconomic variables, higher age was associated with an increased risk of LLA, with risk increasing 8% per year of age at the time of diagnosis, HR 1.08 (95% CI 1.05–1.10). Men had a 1.57 times higher risk of LLA than women, HR 1.57 (1.06–2.34), and being divorced indicated a significant increase in risk compared to being married, HR 1.67 (1.07–2.60). When marital status was separated by gender, divorced men had a significantly increased risk of LLA compared to being married, HR 2.34 (1.42–3.88). Women showed no significant associations regarding marital status (results not reported).

Among medical variables, people treated with insulin had a significantly higher LLA risk than individuals who only had diet treatment, HR 2.03 (1.10–3.74). Worse foot risk groups showed relatively high magnitude associations, although CI tended to be wide: neuropathy/angiopathy, HR 4.12 (2.84–5.98), previous DFU, HR 8.26 (3.29–20.74), and ongoing severe foot disease, HR 11.24 (4.82–26.23).

In lifestyle variables, smoking was associated with an increased risk compared with no smoking, HR 1.99 (1.28–3.09). Regarding activity, low PA defined as <1 time/week was associated with an increased risk with an HR of 2.05 (95% CI 1.10–3.74) compared with daily PA. The only lifestyle variable associated with a statistically significant decreased risk in the adjusted model was obesity, HR 0.46 (0.29–0.75), compared with normal weight.

## 4 DISCUSSION

In this study the results showed that persons who were older, men, divorced, treated with insulin, smoked, had a low PA, and had a higher degree of category 2–4 in the foot risk category had an increased risk for LLA in the adjusted model. Being obese was associated with a decreased risk. These findings indicate that a broader perspective on risk factors might be warranted in people with newly diagnosed diabetes and contribute useful information which can be used to develop early interventions for DFU as well as targeted intervention programs.

### 4.1 Demographic and socioeconomic variables

The duration of diabetes versus higher age has been debated as risk factors for LLA [26]. We found that older age is associated with a higher risk for LLA even in persons with a short duration of diabetes; indicating that older persons with diabetes should receive extra attention even if the disease duration is relatively short. Higher age at diabetes diagnosis was associated with a higher LLA risk. This was expected since higher age has been identified as a risk factor in several previous studies [27]. Previous work has suggested that the duration of diabetes, not age, might be the predominant factor contributing to an increased risk of LLA, demonstrating that a 10‐year longer duration of diabetes was associated with a 2–4‐fold increased risk for LLA [28]. Earlier studies have however included only persons with a long duration of diabetes, making it difficult to differentiate between higher age and a longer duration of diabetes as a risk factor. In the present study, we included newly diagnosed individuals with a mean duration from diabetes diagnosis to amputation of 4 years. Our result indicates that higher age could itself be a risk factor for LLA and independent of other variables we adjusted for, including diabetes duration.

In the present study men were found to have an increased risk of LLA. This is consistent with results from previous studies and might be due to a differentiating approach to care between genders [8, 27, 29]. Women have been shown to be more active in self‐care and preventive care, search for information, and trying to adapt to the situation while men more often seek help for acute problems, have a pessimistic view of the future and tend to have a more passive attitude, although they do tend to discuss foot‐related problems [30]. Combined results suggest that different approaches to care may improve outcomes for men and women and offer evidence for the development of educational initiatives. This will be particularly important for men, who are at an increased risk of LLA.

Persons who were divorced had a higher risk of LLA. This may be due to a change in self‐care and food habits observed in people when they divorce [31]. Previous research has indicated that men in particular have poorer self‐care following a divorce [28]. This finding is likely a combined effect of the general self‐care behavior of men and changes following divorce. Persons who were widowed had a higher risk of LLA in the unadjusted model but not in the adjusted model. In a secondary analysis, we found that this association was nullified in the adjusted model primarily because we adjusted for age (results not reported). This knowledge that a person's social situation affects outcome in relation to LLA indicates the need for further research to identify the underlying reason why people who are divorced are at an increased risk as well as educational efforts targeting people in high risk groups.

In the present study we used education level as an indicator of socioeconomic status. However, education did not show a statistically significant association with LLA in the adjusted model. This finding contradicts results of several earlier studies, which identified an association both between social deprivation level, education level, and risk for LLA among people with diabetes [32, 33]. As stated earlier, the lack of association in our adjusted model does not necessarily indicate that this variable did not play a role in LLA risk, especially as education was associated with LLA in our unadjusted model. The reason the association was nonsignificant in our adjusted model may be that the association of education with LLA was mediated by the medical or lifestyle variables that we adjusted for. It could also be that the Swedish healthcare system, being accessible to all, may mitigate some of the impact of social status on LLA risks.

It has previously been shown that some ethnicities have a greater burden of diabetes than others [12]. In general, ethnicity is not recorded in Swedish registers and our data set only included data on being born in or outside of Sweden. This does not necessarily indicate ethnicity but rather nationality. In our results there was no significant difference in LLA risk between persons being born in Sweden or not. This may be due to the fact that we only had data on country of birth (not ethnicity) and that only 15 persons who had an LLA were born outside of Sweden. Further investigation of this is important and requires longer term follow‐up.

### 4.2 Medical variables

Results indicated that early lower limb complications after a diabetes diagnosis, or complications present at diagnosis, are risk factors, and suggest that these patients should be given extra attention. The finding that treatment with insulin showed a significantly higher risk for amputation, but the combination of insulin and tablets did not, may be related to a more severe disease in those treated with insulin only [34]. It should be recognized however that the latter group consisted of only 9 subjects; therefore, this result warrants further investigation. Several studies have found an association between HbA1c levels and LLA [29, 35, 36]. A meta‐analysis examining the association between HbA1c levels and LLA calculated an odds ratio for LLA incidence as 1.2 for every 1% HbA1c increase [36]. In our analysis, HbA1c was not associated with LLA in either the unadjusted or adjusted model, but the CI marginally overlapped the zero effect. Furthermore, hypertension and GFR were significantly associated with LLA risk in the unadjusted models but not in the adjusted model. This finding may seem to contradict some earlier research showing that hypertension and renal dysfunction increase the risk for LLA [29, 37]. However, our results should be interpreted in light of the other variables included in the adjusted model. These variables may also be more relevant for LLA risk when diabetes duration is longer. The reason they are not associated with LLA in this study might be because only people with newly diagnosed diabetes were included, or that the adjusted method is too conservative when it mutually adjusts for all included variables.

In Sweden most primary care units conduct opportunistic screening for people with increased risk for diabetes. This means that more people receive their diabetes diagnosis early after diabetes debut, resulting in improved glucose control, and a reduced risk for complications [38]. Early detection of diabetes also means that treatment of risk factors such as hypertension, dyslipidemia, metabolic control, etc. can be initiated at an earlier stage, reducing the risk of foot ulcers/amputation [39].

High lipids were not found to be significantly associated with LLA, contradicting an earlier study demonstrating that high lipid levels are a driving factor for neuropathy in persons with type 2 diabetes [40]. Reasons for contrasting results are not clear and more studies investigating this factor are needed. Foot risk factors were a very strong predictor of LLA, with Groups 2–4 (neuropathy/angiopathy, previous DFU, and ongoing severe foot disease) recording a higher risk over other variables in the study. This finding aligns with previous research [15, 41, 42]. It also aligns well with the IWGDF guideline for foot ulcer prevention which has a strong emphasis on foot status [18]. The result emphasizes the importance of addressing persons with these problems with great vigilance to avoid an outcome of LLA [41]. Since these foot risk categories have been shown to carry high financial costs even without amputation, both for the individual and society, focusing on early warning signs could be a cost‐effective measure [5]. It is clear from our material that some patients have a foot risk category of 2–4 at the time of their diabetes diagnosis. This could be related to the common fact that patients often have had diabetes for some time before their diagnosis, and that changes in foot risk factor might have been what triggered the health care provider to consider diabetes as an underlying diagnosis. Foot risk factor 2, neuropathy/angiopathy carried a greatly increased risk of LLA despite being the lowest score on the scale over normal foot health, HR 4.12 (2.84–5.98) in the adjusted model. These are symptoms that are not uncommon to have at the time of a diabetes diagnosis and raises the question of how to reach this patient group before the symptoms lead to the first foot ulcer and an even greater risk for LLA. Some studies have indicated that regular diabetes screening of type 2 diabetes may reduce the time to clinical diagnosis of diabetes and presumably the risk of people already having developed foot complications when diagnosed. On the other hand, studies have shown that earlier diagnoses made possible through screening does not necessarily result in improved outcomes on a group level [43]. It could be that additional factors such as genetics or other, not yet studied factors, also influence outcome regardless of an early diagnosis.

### 4.3 Lifestyle variables

This study indicates that lifestyle variables have a strong association with LLA, and an increase in PA, avoidance of being underweight, and smoking cessation may be impactful interventions to reduce the risk of LLA. In our study, smoking was associated with an increased risk of LLA even after mutually adjusting for various variables. This finding was in line with previous studies [13, 29, 42]. The decline in the incidence of LLA in previous studies could be due to the association with smoking and that smoking is becoming less prevalent in the Swedish population. Even though there is a slightly decreasing trend for people <60 years old with diabetes, 18.7% of men and 15.9% of women still smoke [38, 39, 44].

The results of this study showed that daily exercise, reported at the start of follow‐up, was associated with a lower risk of amputation compared to PA less than once per week. While PA has been increasing in Sweden in the general population we have seen very little change in the Swedish population with diabetes between 2002 and 2021 [39, 45]. Traditionally, too much standing and walking has been thought to increase the risk for DFU in individuals with diabetes, but more recent research tends to consider standing and walking safe and perhaps even beneficial if appropriate offloading devices are used [46]. In Sweden, there is a possibility for health care providers to prescribe PA as a complement to other interventions. The effect of PA on ulcer healing has been studied earlier in a few studies, but the effect on persons with diabetes and the risk for amputation would be an interesting subject for future studies [45, 47, 48].

An increased BMI has been previously associated with a decreased risk for amputation, and very low BMI is a risk factor for LLA [42]. In our adjusted model, among lifestyle variables, only obesity correlated with a lower risk of LLA. This finding might be exaggerated by more comorbidities and increased risk for those with low BMI, that is, some people of normal weight may have lost weight due to illness (reverse causality). All lifestyle variables (BMI, smoking and PA) were associated with LLA in the model adjusted for several other factors. This finding highlights the importance of lifestyle variables in association with the risk of LLA and should urge us to continue educational efforts promoting the importance of lifestyle choices for people with diabetes, including regular PA, avoiding being underweight, and smoking cessation. The fact that obesity was associated with lower risk for LLA presents itself as a conundrum regarding how to relay this information to patients considering the many well‐established risks associated with obesity.

### 4.4 Strengths and limitations

A strength of this study is that only people with recently diagnosed diabetes were included, meaning that we analyzed data from early disease progression, which could provide insight into variables that are early warning signs for a future risk of LLA. By following individuals with newly diagnosed diabetes, we also obtained a cohort that has a comparable duration of diabetes, which is a variable that otherwise may overshadow other risk factors [28]. Further strengths of this study are that it is based on register data with a high coverage in the general Swedish population and the possibility to cross‐reference it with national registers covering demographic and socioeconomic, medical, and lifestyle variables and due to the compulsory nature of those registers the risk of selection bias is minimized.

A limitation of the analysis is that analyzed individuals must have complete data on the relevant variables even after removing variables with missingness higher than 40%, which led to a large number of excluded participants. Some missing data likely comes from patients not attending annual check‐ups, and there can also be missing data when variables are not all filled in at annual check‐ups. We cannot know if missing data were random, and thus, the risk of biased estimates cannot be excluded. As we mutually adjusted for factors simultaneously, some variables may have been over adjusted. Therefore, variables that did not show an association in the adjusted model should not be ruled out as potential risk factors for LLA [49]. However, the variables that retained statistically significant associations with LLA in such an extensively adjusted model may play a role in the risk of LLA among individuals with diabetes. We were also limited in the choice of variables to those included in the NDR. As a result, there are potentially relevant risk factors that may have been overlooked, such as barefoot walking and inappropriate footwear. The variable foot risk factor used by NDR lacks guidelines for classification by the healthcare professionals, potentially affecting validity of the scale negatively. Another limitation was that we did not have access to data on macrovascular complications at the time of diagnosis.

The results of this study primarily reflect the situation in Sweden. Sweden has a public healthcare system with access to care and medicines for everyone regardless of financial or social status. This limits generalizability of results to other healthcare contexts. Sweden does however have high quality national registers, which is a strength that should make some conclusions more generalizable. Furthermore, due to the general nature of many of the included risk factors, the results should in large parts be generalizable to other countries.

In order to bring about change in diabetes care it will be important to integrate new knowledge of risk factors into patients' daily care. Future research should focus on developing and evaluating personalized education efforts based on specific risk factors (e.g., age, male sex, divorce, and BMI) as well as evaluating additional risk factors which present early in the disease progression and are strongly associated with LLA. Further quantitative and qualitative studies into effective approaches and interventions for specific patient groups is warranted.

### 4.5 Conclusion

This study found a higher risk for LLA among people with a higher age, men, people who were divorced, who had a higher foot risk group, lower PA level, who were on insulin treatment, and who were smokers. Obesity was associated with a lower risk for LLA. These variables may have important roles in LLA risk among individuals with diabetes.

We found no significant association between risk for LLA and education level, country of origin, type of diabetes, blood glucose level, hypertension, hyperlipidemia, creatinine level, or GFR.

### 4.6 Clinical implications

Lifestyle variables have a strong association with LLA, and an increase in PA, avoidance of being underweight, and smoking cessation may be impactful factors in reducing the risk of LLA. While education on the risks of LLA is common, our results suggest that efforts may be more effective if they are adapted to address the needs and characteristics of specific patient groups.

Early lower limb complications after a diabetes diagnosis, or complications present at diagnosis, are strong risk factors and these patients should be given extra attention. The strong positive association between LLA and foot risk category 2–4 at time of diagnosis should be clearly communicated to patients and health care providers.

The duration of diabetes versus higher age have been debated as risk factors for LLA. We found that older age is associated with a higher risk for LLA even in persons with a short duration of diabetes. Older persons with diabetes should receive extra attention regarding preventive care even if the disease duration is relatively short.

Combined with future research findings, knowledge from the present study may contribute to policy decision making and guide national funding bodies who support patient education and prevention initiatives.

## AUTHOR CONTRIBUTIONS

**Simon Ramstrand**: Formal Analysis; project administration; funding acquisition; writing—original draft; writing—review & editing. **Michael Carlberg**: Data Curation; formal analysis; funding acquisition; methodology; writing—review & editing. **Gustav Jarl**: Conceptualization; funding acquisition; project administration; supervision; writing—review & editing. **Anton Johannesson**: Conceptualization; funding acquisition; writing—review & editing. **Ayako Hiyoshi**: Conceptualization; data curation; funding acquisition; writing—review & editing. **Stefan Jansson**: Conceptualization; funding acquisition; writing—review & editing.

## CONFLICT OF INTEREST STATEMENT

The authors declare no conflicts of interest.

## ETHICS STATEMENT

The study was approved by the regional ethics committee review board in Uppsala, Sweden (reg. no. 2017/533 and 2017/533/1).

## Supporting information

Supporting Information S1

## Data Availability

The data that support the findings of this study are available from the National Board of Health and Welfare, Statistics Sweden and the Swedish National Diabetes Register, but restrictions apply to the availability of these data, which were used under license for the current study and so are not publicly available.
