# Supplementary material for: Exploring potential risk factors for lower limb amputation in people with diabetes—A national observational cohort study in Sweden
Source: J Foot Ankle Res. 2024 Sep 1;17(3):e70005. doi: 10.1002/jfa2.70005 (PMC11366273; doi:10.1002/jfa2.70005)
Supplement: Supplementary file 1 — Supporting Information S1 [file JFA2-17-e70005-s001.docx]

# Supplement A - The Swedish National Diabetes Register (NDR)

The Swedish National Diabetes Register, NDR, was launched in 1996 for the purpose of promoting evidence-based development of diabetes care by offering up-to-date information about changes in the treatment of glycaemia and other risk factors, as well as diabetic complications. Another aim is to support improvement in the quality of care provided by participating units at hospitals and primary care clinics. The overall objective is to reduce morbidity and mortality, as well as to maximize the cost-effectiveness of diabetes care.

All variables included in NDR are listed below.

| Acanthosis nigricans |
| --- |
| Albuminuria |
| Proportion (%) of time with glucose values within the range 4-10 mmol/L in rtCGM/isCGM (time in range, TIR) in the last 2 weeks. |
| Proportion (%) of time with glucose values within the range of 4-8 mmol/L in rtCGM/isCGM (time in target, TIT) in the last 2 weeks. |
| Proportion (%) of time with glucose values below 4 mmol/L in rtCGM/isCGM in the last 2 weeks. |
| Number of units (U) of basic/long-acting insulin per day |
| Antihypertensive drugs |
| Uses carb counting |
| ASA or other thrombus aggregation inhibitor |
| Treated for eye complication due to diabetes during the last year |
| Date of visit |
| Bloodketones (highest value) |
| Bloodpressure assessment |
| Bloodpressure diastolic |
| Bloodpressure systolic |
| BMI |
| Living in Sweden at diagnosis |
| C-peptide |
| Celiac disease |
| Cerebrovascular disease ever |
| Diabetes treatment |
| Diabetic retinopathy |
| Type of diabetes |
| Type of diabetes visit |
| Date of diagnosis |
| Year of diagnosis |
| Visit/appointment |
| Remote visit |
| U/KG/day |
| Self-care plan drawn up date |
| Foot examination during the last year |
| Foot examination, date |
| Physical activity |
| Physical activity - 30 min walk or equivalent |
| Date of birth |
| GAD |
| GFR |
| HbA1c |
| HDL |
| Hypoglycaemia incidence severe - help from another person, numbers during last year |
| IA2, RBA |
| IAA |
| Indication for pump treatment |
| Internal Patient ID |
| Ischemic heart disease ever |
| IsoBMI |
| Ketoacidosis (how many since last visit) |
| Cholesterol |
| Sex |
| Laser or other treatment, during last year |
| LDL |
| Lipid-lowering drugs |
| Lowest pH |
| Height |
| Average glucose in rtCGM/isCGM last 2 weeks. |
| Method of giving insulin |
| P-glucose (highest value) |
| P/S creatinine |
| Patient deceased |
| Social security number |
| Polydipsia |
| Polyuria |
| Pump treatment terminated, state main reason |
| Ongoing pump |
| Retinopathy, diagnosis in the most affected eye |
| Risk category foot |
| Smoker |
| Smoking habits |
| S-free-T4 |
| S-IgA gliadin antibodies |
| S-IgA transglutaminase antibodies |
| S-IgG-gliadin antibodies |
| S-IgG transglutaminase antibodies |
| S-TSH |
| Sensor-based continuous glucose measurement (rtCGM/isCGM) |
| Serial number running pump |
| Quit smoking year |
| Quit snuff year |
| Snuff habits |
| Standard bicarbonate (lowest value) |
| Standard deviation (SD) mean glucose in rtCGM/isCGM last 2 weeks. |
| Severe hypoglycaemia, unconsciousness with or without convulsions. presence since last visit. |
| Visual impairment due to diabetes |
| Thyroid disease |
| Total dose of insulin per day |
| Triglycerides |
| Equipment Type (rtCGM/isCGM) |
| Thyroxine treatment |
| U-Albumin/Creatinine |
| Weight |
| Weight loss |
| ZnTBA |
| Age |
| Islet cell |
| Eye fundus examination, date |

# Supplement B - Longitudinal integrated database for health insurance and labour market studies (LISA)

Statistics Sweden’s longitudinal database, LISA, comprises detailed data on health insurance, parental insurance, and unemployment insurance at the individual level. LISA enables the study of individuals’ transition over time between, for instance, gainful employment, unemployment, and illness.

Most variables in the administrative registers apply a whole year as a measurement period, using the population on 31 December. Variables that indicate employment use the month of November as a measurement period, while the highest level of education completed refers to the spring term and study attendance refers to the autumn term.

The variable content in LISA is grouped mainly by:

- Demographics
- Education and training
- Employment and unemployment
- Income and social insurance
- Family
- Local unit and enterprise

# Supplement C – In-Patient Register

The In-Patient Register is part of the National Patient Register and provides the basis for statistics on diseases and treatments in Swedish specialized care.

The Patient register contains information on:

- All completed inpatient stays since 1964 (nationwide since 1987)
- Data on patients treated by doctors in specialized outpatient care since 2001.
- Data on patients admitted to compulsory psychiatric care under the Compulsary Psychiatric Care Act since 2010
- Data on waiting times at emergency departments since 2016.

The information we used in this study from NPR were:

**Patient Data**

• Personal registration number

• Gender

• Age

• Main Diagnosis

• Secondary Diagnosis

• External Cause of Injury and Poisoning

• Procedures

# Supplement D – The Total Population Register (Statistics Sweden)

Population statistics show population size and population changes, such as the number of births, deaths, and immigration and emigration and are available by county, municipality, sex, age, civil status, country of birth and country of citizenship. The statistics are based on data on registered persons that the Swedish Tax Agency supplies to Statistics Sweden.

We used this register to track emigration and death.

Supplement E - Nordic Classification of Surgical Procedures

We used the following amputation codes:

1. high proximal amputations:

NEQ19 Transpelvic amputation

NFQ09 Transfemural amputation

NFQ19 Hip disarticulation

NGQ09 Knee disarticulation

1. low proximal amputations:

NGQ19 Transtibial amputation

NHQ09 Talocrural disarticulation

NHQ11 Talocrural amputation

1. partial foot amputations:

NHQ12 Transtarsal amputation

NHQ13 Tarsometatarsal amputation

NHQ14 Transmetatarsal amputation
